# Effect of an Intervention Program Based on Active Video Games and Motor Games on Health Indicators in University Students: A Pilot Study

**DOI:** 10.3390/ijerph15071329

**Published:** 2018-06-25

**Authors:** Félix Zurita-Ortega, Ramón Chacón-Cuberos, Manuel Castro-Sánchez, Francisco Luis Gutiérrez-Vela, Gabriel González-Valero

**Affiliations:** 1 Department of Didactics of Musical, Plastic and Corporal Expression, University of Granada, 18071 Granada, Spain; felixzo@ugr.es; ggvalero@ugr.es; 2 Department of Integrated Didactics, University of Huelva, 21007 Huelva, Spain; ramon.chacon@ddi.uhu.es; 3 Department of Education. University of Almería, 04120 Almería, Spain; mcastros@ual.es; 4 Department of Languages and Computer Systems, University of Granada, 18071 Granada, Spain; fgutierr@ugr.es

**Keywords:** video games, motor games, physical fitness, Mediterranean diet, resilience

## Abstract

(1) Background: High levels of physical inactivity caused by sedentary digital screen leisure constitute one of the main causes of the high levels of obesity observed in today’s society; (2) Methods: The present study aims to analyse the effect of a 12-week intervention program based on the application of active video games and motor games on health status indicators, problematic use of video games, and resilience capacity in university students. Besides, the content blocks of the Physical Education (PE) field are worked on through these devices, revealing their potential as an Information and Communications Technology (ICT) resource. A longitudinal study with a pre-experimental design with pretest–posttest measurements in a single group (*n* = 47) was performed, using as main instruments a Tanita TBF300® bioimpedance scale, the 20mSRT test for maximum oxygen consumption (*V*O^2^_max_), the Adherence to a Mediterranean Diet Test (KIDMED), the Questionnaire for Experiences Related to Video games (QERV) and the Connor-Davidson Resilience Scale (CD-RISC); (3) Results: The main results were a discrete improvement in the percentage of fat mass and *V*O^2^_max_, representing a small effect size in both cases. The quality of the diet followed and the confidence and tolerance for adversity as a resilience factor were also improved, representing a medium size effect for this last variable; (4) Conclusions: Despite the limitations of this study as it does not have a control group, the main conclusions are that active video games and motor games can be a motivational resource to follow an active lifestyle, helping to improve health status indicators in young adults.

## 1. Introduction

### 1.1. Theoretical Framework

University stage is defined as a critical risk period regarding maladaptive behaviour which is harmful to human health [1]. This period takes place in emerging adulthood defined by Arnett [2] and it is characterised by the young adults’ engagement in higher education [3]. These young people begin to abandon adolescence, since they acquire roles typical of adults which give them autonomy, having to initiate themselves into the labour world in order to self-finance themselves or having to leave their family life [1,2]. However, they have not yet acquired full socio-emotional maturity, being common the development of self-harmful behaviour such as using legal drugs, not following a high quality diet or having the habit of using sedentary digital leisure, thus leading to high indices of overweight [4].

Problematic use of video games is presented as a risk factor in the development of health problems at a physical and cognitive level in university students [5,6]. Regarding negative impact in the context of health, scientific production has been related to the inverse relationship between frequency of use of video games and the decrease in levels of Physical Activity (PA), increasing levels of sedentariness and obesity [7,8]. This is associated with an increase in fat mass and a decrease in lean mass, a worse maximum oxygen consumption (*V*O^2^_max_), deteriorated physical abilities or deteriorated psychosocial factors such as self-esteem [9,10].

This situation is promoted by high intake of junk food or poor quality diets followed currently [11], which stand out because of their high amounts of sugar, fat, additives and salt [11,12]. In light of this situation, several authors propose promotion of the Mediterranean Diet (MD), which enables a healthy lifestyle. This diet is characterised by the consumption of typical food from the Mediterranean Region, standing out are the high intake of natural antioxidants, legumes, fruit and vegetables, fish, dried fruit and nuts and olive oil [13]. Following a healthy diet together with an active lifestyle enables not only improvement of physical health but also of specific psychosocial factors such as self-concept, self-esteem or resilience [13,14].

Specifically, resilience is defined as a process through which individuals use personal and environmental elements in order to adapt themselves or redirect traumatic and stressful elements in their daily life [15]. In this sense, resilience capacity will integrate cognitive and affective components, as well as abilities and behaviour enabling the development of healthy behaviours and positive answers in facing adversity, increasing the life expectancy of people [16]. Numerous studies have demonstrated that this has a close relationship with the practice of physical activities and sports, being a plenty valuable resource for its development [17,18].

### 1.2. State of the Question

Facing the problem of high indices of overweight produced by physical inactivity, sedentary digital leisure and little adherence to Mediterranean Diet, various authors emphasise the importance of promoting physical-healthy habits based on the practice of hedonistic physical and sporting activities with the aim of creating intrinsic motivations towards these kind of behaviours [4]. Active video games constitute a booming resource to achieve these goals [19]. These are those video games which enable the transfer of the player’s corporal movements to virtual reality which can be observed on the screen by means of different peripherals [20]. There is a clear connection between these devices and the practice of physical activity, since studies such as the ones by Oh & Yang [20] and Kahlbaugh et al. [21] establish that the use of these devices will imply physical effort, which will depend on the physical exercise entailed by the video game. Based on all this, this kind of video games will represent a kind of active leisure which could help improve certain values indicating health, this being demonstrated by Agmon et al. [22] or Sun [23].

In this sense, several studies with similar characteristics have been those carried out by López-Sánchez et al. [24] or Borrego et al. [25], which show how an intervention program based on moderate physical activity can improve different health indicators such as body composition or maximum oxygen consumption (*V*O^2^_max_). Addressing researches done with active video games, Maddison et al. [26] demonstrated how a program based on active video games over 24 weeks decreased the fat mass of the experimental group, also decreasing sedentary levels. This is due to the increase of time spent in the use of these devices at home by the respondents. In a similar way, Gribbon et al. [27] developed an intervention study in which they verified the effect of the use of the Kinect platform over one hour daily in energy expenditure, concluding an increase of this alone in the first 24 hours. Finally, it should be noted that these devices have been used even in other populations such as adults, helping to improve the level of mobility, balance and functional capacity [28].

This research study aims at developing a pilot study based on the application of active video games and motor games in a sample of university students in order to check their effectiveness in the improvement of parameters indicating health levels, with the aim of applying similar processes in school-age children in the future. The sample is made up of future Physical Education (PE) teachers and the interest of the study is based on what is established by the Law of 17 March 2017 which formulates the curriculum corresponding to Primary Education in Andalusia (Spain) which specifies in aim number 7 of the Physical Education subject the importance of using Information and Communications Technology (ICT) as an additional support [29,30]. Specifically, the proposed intervention program is performed in 12 weeks with the aim of working for three weeks on each of the four content blocks for Physical Education (PE) at the same time as achieving a minimal effect in the studied parameters [29].

Considering the premise of the existing problem of high sedentary and obesity indices, it can be established that these are propitiated by low levels of physical activity, high rates of sedentary digital leisure and a poor quality of the diet in emerging adulthood. This study presents the following research question in order to address the need to generate alternatives to set an active lifestyle that enables improving health in youth and then to be transferred to lower stages to work with PE content as suggested in previous research [31,32]: Could an intervention program based on active video games improve different health indicators such as body composition, *V*O^2^_max_ or adherence to the Mediterranean diet?

Following the problems raised and the need to act before it, these hypotheses are proposed in relation to the intervention program developed through active video games:

- Hypothesis 1 (H1): The basic descriptive will show a higher prevalence of lean mass and *V*O^2^_max_ in men. Women will present greater flexibility and adherence to the Mediterranean diet. The problematic use of video games will be more widespread in men. Women will be more resilient.
- Hypothesis 2 (H2): The intervention program will improve the percentage of fat and lean mass, *V*O^2^_max_ and the flexibility of university students.
- Hypothesis 3 (H3): After the intervention program the problematic use of video games will decrease and it will improve the levels of resilience and adherence to the Mediterranean diet.

Therefore, this study pursues as main goals to: (a) Set the percentage of fat mass and lean mass, flexibility, *V*O^2^_max_, adherence to MD, problematic use of video games and resilience in a sample of university students of PE depending on their gender; (b) Verify the effect of an intervention program based on motor games and active video games in the described variables; (c) Analyse the connection between these variables after the performance of the intervention program.

## 2. Materials and Methods

### 2.1. Subjects and Design

The intervention was performed on a natural group of students of the university degree in Primary Education, therefore creating a pre-experimental design in randomised natural groups—Cluster-Randomized Controlled Trial [33]. In this sense, a longitudinal study was carried out with a pretest–posttest design with a single group without control group. The design is unifactorial and multivariate having only one independent variable—group—and diverse dependent variables—fat mass, lean mass, flexibility, et al. The sample was made up of 47 participants, being 61.7% men (*n* = 29) and 38.2% women (*n* = 18). The average age was 22.53 (*SD* = 2.19), with a minimum age of 20 and a maximum age of 28. The selection of participants was made on convenience. As inclusion criteria, it was considered that respondents were student of the last year of the degree in Primary Education, as well as not suffering any diseases which could hinder the normal progress of the program. The previous play experience with active video games was not considered.

### 2.2. Measures

Height was calculated using a SECA-213 stadiometer® (m).

Body weight (kg) and Body composition—percentage of fat mass (%) and lean mass (%)—was established by means of electronic weighing scales, using the model Tanita TBF300®. This model needs the variables of gender, age and height, measuring this last one by means of a stadiometer Holtain LTD® and following the protocol established by Portao et al. [34].

Body Mass Index (BMI) was calculated from the arithmetic ratio established by Quetelet [35]—body weight/height^2^ (kg/m^2^).

The *V*O^2^_max_ was measured indirectly with the test “Meter Shuttle Run Test (20mSRT) [36]. This test is of maximum incremental character and consists in running a round distance of 20 meters following the speed set by the protocol 20mSRT. The initial speed set by rate is 8 km/h, increasing 0.5 km/h each minute. In order to calculate *V*O^2^_max_ indirectly we used the speed reached in the last stage, using the following formula: *V*O^2^_max_ (ml/min/kg) = (6 × FA) − 27.4 [37,38].

Flexibility was assessed by means of the sit and reach flexibility test for the upper body [39]. The protocol consists in moving a cardboard box situated in a straight line parallel and adjacent to the person’s heel without moving the feet and allowing bending of the knees. The final position must be kept and held while measuring the distance reached in centimetres (cm).

Adherence to a Mediterranean Diet, assessed by the questionnaire KIDMED [40]. This test is made up of 16 dichotomous items with an affirmative or negative answer (Ex: 1. You eat a fruit or drink a natural fruit juice every day), which refer to patterns related to the Mediterranean model. Four of these items have negative connotations (−1), whereas the other twelve are positively valued (+1), ranging the final score from −4 to +12. This questionnaire scored an internal consistency of α = 0.77.

Questionnaire of Experiences Related to Video games (QERV), validated by Chamarro et al. [41] for teenagers. This test assesses the problematic use of video games and is made up of 17 items with negative connotations (Ex: 1. To what degree do you feel restless facing issues related to video games?), which are valued by means of a Likert scale with four options (1 = Hardly ever; 2 = Sometimes; 3 = Quite often; 4 = Almost always). This instrument enables the assessment of problematic use of video games using a summation which classifies the variable in terciles. The reliability of this instrument was α = 0.89.

Connor–Davidson Resilience Scale (CD-RISC) [42], for the assessment of resilience in each individual. It is made up of 25 items (Ex: 1. I am able to adapt to changes); the person has to decide the degree to which each statement is true for him or her in the last month. A Likert scale is used ranging from 0–4 where 0 represents “I totally disagree” and 4 “I totally agree”. Factoriality of the items forming this instrument enables the creation of five dimensions associated to resilient behaviour, such as 1 = Personal ability and Tenacity; 2 = Confidence and tolerance for adversity; 3 = Positive acceptance of change; 4 = Control; 5 = Spiritual influence. This instrument achieved an acceptable reliability of α = 0.83.

Ad Hoc questionnaire for the record of socio-demographic variables (gender, age, place of residence, etc.). An item was also included in order to know if the participants suffered any kind of disease which could hinder the involvement in the study.

### 2.3. Procedure

In the first place we asked for the licences needed and the informed consent. Approval of the research by the Human Research Ethics Committee of the University of Granada was requested, being sanctioned with code 462/CEIH/2017. Regarding participants, all of them were adults. An informative letter was provided describing the main characteristics of the study, ensuring anonymity of data and its scientific research purposes. The sample for the study was made up of those participants who decided to take part in it signing the informed consent.

The study was accomplished from October to December 2016. For its execution, researchers performed two formative sessions where they detailed the characteristics of the intervention plan, as well as the tasks to be performed by each member of the project. Periodicity of intervention consisted of two weekly sessions lasting two hours each, which were divided into four phases.

Concerning phases in each session, the first phase (1) consisted of a short introduction explaining the contents that were going to be worked on lasting about 10 minutes. Arrangement of the working sessions was aimed at working on all of the four educational content blocks defined by the LOMCE [30], lasting for 3 weeks each. During the second phase (2) a global warm-up exercise was performed running for about 10 minutes in order to move joints and activate muscles. The third phase (3) represented the main phase of training lasting for one hour and a half. This period is divided into two parts of 45 minutes each, where the experimental group is divided. During the first part a subgroup works on the corresponding contents using motor games, while the rest work on those same contents using active video games. Later, roles are inverted in order to make both subgroups work the same amount of time with both methods. The fourth phase (4) consists in recovering after exercise by playing motor games with a lower internal load aiming at favouring recovery of the normal functions of the body.

**Figure 1:**
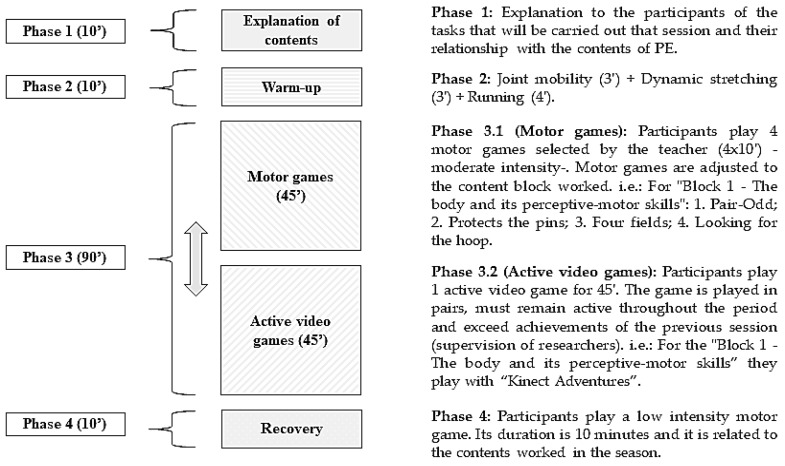
Explanation of a session of the intervention program.

Motor games used during phase 3 were brought together following the content blocks in current legislation [29,30]. In the same way, active video games were used in order to work on those contents in the corresponding subgroup. For this, Xbox 360® platform was used using the motion sensor Kinect®. For “Block 1: The body and its perceptive-motor skills” the video game “Kinect Adventures®” is used, for “Block 2: Physical Education as a health improver” the video game “Kinect Training®” is used, for “Block 3: Body language” “Kinect Dance Central®” is used, and for “Block 4: Play and sport in schools” “Kinect Sports®” is used (Figure 1).

**Figure 2:**
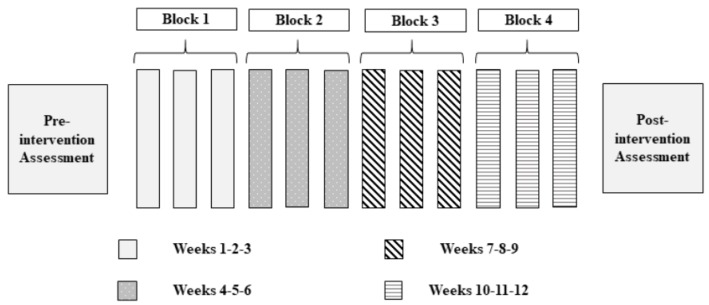
Intervention program based on active video games and motor games (weeks).

It was expected to obtain a similar internal load through motor games and active video games, since as Miyachi et al. [43] state, these can involve physical activity of a moderate intensity. The splitting of the group was done as a result of the impossibility to work with active video games with the whole group at the same time, due to the high amount of devices required. The arrangement of the intervention sessions is shown graphically in Figure 2.

We have to emphasise that the intervention program was designed and analysed thoroughly regarding the mentioned characteristics, being created and supervised by professional researchers in the fields of Physical Education, Physical Activity and Sports Sciences and ICT. In the same way, we have to point out that this research study followed the ethical principles for research established by the Declaration of Helsinki. The participants’ right to confidentiality was also observed at all times.

### 2.4. Data Analysis

Statistical analysis was carried out using the software IBM SPSS® 22.0 (IBM Corp, Armonk, NY, USA). For basic descriptors, frequencies and medians were used, whereas for the study of connections between variables a T-test was used with independent samples and bivariate correlations of Pearson. In order to analyse the effect of the intervention program we used a T-test of related samples and Cohen’s d together with the confidence interval (95%) [44]. Normality of data was checked by Kolmogorov–Smirnov’s test, using Lillieforts’ correction and homoscedasticity using Levene’s test. Internal reliability of the instruments used was valued using Cronbach’s Alpha coefficient, fixing a Reliability Index of 95.5%.

## 3. Results

Table 1 shows basic descriptors of the sample studied regarding gender of participants. Statistically significant differences were observed in the scores obtained between men and women for body weight (74.87 ± 11.21 vs. 55.79 ± 8.11) and BMI (24.08 ± 3.70 vs. 21.09 ± 3.41). A higher median of fat mass was found in women not being statistically significant (12.75 ± 5.91 vs. 12.00 ± 5.56), whereas regarding lean mass statistically significant differences were found with a higher average percentage in men (62.84 ± 6.55 vs. 43.03 ± 2.78). Flexibility established by deep flexion of the upper body revealed a value of 28.91 cm for men and 29.96 for women not finding statistical differences. In relation to maximal oxygen consumption (*V*O^2^_max_) statistically significant differences were found, being higher in men (51.12 ± 12.79 vs. 38.54 ± 12.56). The degree to which a Mediterranean diet is followed did not reveal statistical differences regarding gender, although men followed a healthier diet (6.68 ± 2.17 vs. 6.09 ± 2.81). Finally, statistically significant differences were found in the problematic use of video games, being greater in men (35.94 ± 11.01 vs. 24.52 vs. 5.43).

Next, the scoring of the different factors forming resilience is shown (Table 2). In this case, no statistically significant differences were found in any dimension, men achieving higher average values in all of them except in “Control” and “Spiritual influences”.

Table 3 shows the effect of the intervention program on the different variables studied. Statistically significant differences are found between data obtained in the pre-test and the post-test for lean mass (*p* = 0.015), the *V*O^2^_max_ (*p* = 0.008), factor 2 of resilience—confidence and tolerance for adversity— (*p* = 0.001) and the quality of the diet followed (*p* = 0.024). For fat mass a lower average value was obtained after the intervention (12.30 ± 5.67 vs. 11.83 ± 5.28), representing a small effect size (d = −0.11). In the case of *V*O^2^_max_ higher average values are achieved in the post-test (45.91 ± 15.03 vs. 47.19 ± 13.53), representing a small effect size (d = 0.13). Factor 2—confidence and tolerance for adversity—improved after the application of the program (3.00 ± 0.33 vs. 3.14 ± 0.38), representing a medium effect size (d = 0.42). Finally, scores obtained in the following of the Mediterranean Diet improved in the post-test (6.44 ± 2.44 vs. 7.11 ± 2.20), representing a small–medium effect size (d = 0.32).

Table 4 shows correlations between variables studied after the intervention. Regarding fat mass there are significant connections with flexibility, *V*O^2^_max_ and the adherence to a Mediterranean Diet, showing a negative and indirect relation in every case (r = −0.296; r = −0.476; r = −0.262, respectively). Regarding lean mass, it correlated positively and directly with *V*O^2^_max_, resilience ability and problematic use of video games (r = 0.323; r = 0.314; r = 0.424), obtaining for all of them statistically significant differences. Finally, a positive and direct relation can be seen between *V*O^2^_max_ and adherence to a Mediterranean Diet (r = 0.291).

## 4. Discussion

This research study analyses the effect of an intervention program based on active video games and motor games in a sample of university students. The program lasted twelve weeks, working on the four content blocks of the Physical Education area. In particular, it was aimed at the improvement of health status indicators such as percentages of fat mass and lean mass or the *V*O^2^_max_, the problematic use of video games, quality of the diet and resilience. Other similar studies were carried out by Falbe et al. [7], Kahlbaugh et al. [21], Sun [23], Foley et al. [45], Graf et al. [46] or Trost et al. [47], all of them aimed at verifying the effect of the use of active video games in different populations.

The pre-test measurement showed that fat mass was greater in women, whereas lean mass was higher in men. These results seem reasonable given women’s physiological features and the preparation of their bodies for fertility through the release of oestrogens, whereas men have greater muscle mass due to androgenic hormones [48]. Additionally, women were more flexible while men had a higher *V*O^2^_max_. These results are similar to those presented by Carrick–Ranson et al. [49] or Zurita [50], who point out that women are more flexible due to their greater amount of oestrogens, which favour fluid retention thus having their connective tissue lower density, besides a lower lean mass. Likewise, men tend to have a higher *V*O^2^_max_ due to their greater systolic volume, lean mass and haemoglobin concentration [49].

It was also observed that scores obtained by problematic use of video games were higher in men as Espejo et al. [51] showed. The main reason is the greater brain activation in men due to stimulation by rewards, which is related to extrinsic motivation and higher levels of satisfaction connected to the seratonin–dopamin system [52,53]. Likewise, and regarding resilience, the most influential factors were personal ability and positive acceptance of change, without differences regarding gender. These results could be caused due to the small size of the sample studied, since Denovan & Macaskill [17] and Liu et al. [15] find women globally more resilient, although men have a greater control and compromise or tolerance for adversity.

Analysing the effect of the intervention program, a reduction of the percentage of fat mass was disclosed, representing a small size effect. In a similar way Foley et al. [45] obtained a fall of the percentage of fat mass in an intervention performed by means of active video games on teenagers, with special emphasis on girls since they are less physically active. Although the improvements are not relevant, they must be considered since internal load regarding the use of these kinds of games is not very high either—following low-moderate intensity according to Sun [23], and that this intervention was performed in young adults who are physically active, therefore the effect being lower. In addition, Staiano et al. [54] obtained slightly higher results in the loss of body fat in a study conducted in adolescents with obesity. In this case, greater losses were obtained since the population studied started from a worse state of health, although these differences only occurred in young people who attended 75% of the sessions and exceeded at least 2500 steps per session. Thus, active video games can be an effective mean for the loss of body fat, being inefficient for the gain of lean mass as shown by LeBlanc et al. [55]. This is because these devices do not allow working with high loads or high concentric speeds [24,55].

Regarding *V*O^2^_max_, post-intervention assessment displayed a slight improvement finding statistically significant differences. This demonstrates that the combination of active video games and motor games of a moderate intensity enables the improvement of physical shape through aerobic capacity, verified by Bethea et al. [56] in an intervention program carried out on children lasting from 12 to 30 weeks. Specifically, the confidence interval obtained shows that improvements could reach an effect of moderate size, which could be achieved by raising work intensity. It is evident that although work intensity might not be sufficient to improve substantially *V*O^2^_max_, it can generate some of the main modifications which could trigger its improvement, such as cardiac output, cardiac capillary density or alveolar gas exchanges [46,56,57]. In a similar line, Roopchand–Martin et al. [58] obtained an improvement in *V*O^2^_max_ in a pilot study conducted in female university students using XBOX Kinect® even with a similar training volume (between 30–60 minutes three times per week), justifying the findings found. Nevertheless, it is important to highlight that Roopchand–Martin et al. [58] used dance video games that involve a greater percentage of *V*O^2^_max_ than those employed in this study for some content blocks (Blocks 1, 2 and 4). Therefore, the importance of regulating the times, load and type of video game in the use of these platforms is emphasized to obtain a greater effect on health indicators [45,55,58].

The program improved the quality of the diet followed by the students, representing a small–medium effect size. Although nutritional contents were not specifically worked on, Schneider et al. [59] show how the practice of sports can act as an intrinsic motivational factor in order to take care of one’s diet. The studies conducted by Chaput et al. [60] or Chaput et al. [61] analysed the response of food intake to the realization of physical activity by active video games in adolescents, concluding that after its realization it developed positive effects in the energy balance making the participants hungrier but without obtaining significant differences in the intake of macronutrients. These premises, with that established by Schneider et al. [59], could explain the improvement of adherence to the Mediterranean diet observed in this study due to the greater demands of essential nutrients caused by moderate physical exercise [40,61].

Among resilience factors, the program improved confidence and tolerance for adversity, having a medium effect size. Studies such as those by Hartfiel et al. [62] demonstrate how recreational activities which produce satisfaction and comfort can help to overcome periods of adversity. In this sense, hedonistic practice of sports such as that offered by active video games can help to improve resilience factors [17,18]. Although no studies were found that used active video games to improve resilience, Lyons et al. [63] demonstrated how the use of these platforms imply greater levels of fun and engaging in the practice of physical exercise, which would help to increase the confidence levels of the subjects [15,63,64].

It is important to point out some of the reasons why the intervention program has not had the expected effect with some variables. First, the sessions made by active video games did not improve the percentage of lean mass. This may be due to the fact that the internal load generated by this exercise is insufficient to generate muscle adaptations such as the increase in muscle cross section or the thickness of type II fibres. For this purpose, it would be necessary to structure a training program with greater intensity based on a structural orientation [65]. Nor was the flexibility improved, which seems reasonable given that the Range of Motion (ROM) of specific joints was not worked on specifically. Exercise levels were simply increased, which could help increase the ROM by eliminating fat mass acting as a lock [55], although the reduction was insufficient. In addition, the problematic use of video games was not improved. It seems that when the use of these video games becomes pathological, it is not enough to promote active habits that substitute them. In these cases, it is necessary to develop intervention programs which are aimed at behaviour modification [41].

Finally, it is interesting to point out the main limitations of this research study. Among them, we can highlight the use of a pre-experimental design and the non-use of a control group, which would have helped to control more effectively the effect of the program, eliminating the effect of certain external variables. Regarding design, we must stress the use of a single experimental group instead of three experimental groups, considering a Group1 which received the effect of the program through motor games, a Group2 through the use of active video games and a Group3 through the blending of both; this will be considered in the future, as this study has been a pilot experience with active video games. In fact, a combination of both is used since the number of platforms Xbox 360® was not enough. Another limitation to bear in mind regarding the effect of the program is the fact that it has been performed on university students that follow a relatively active lifestyle. Likewise, it would have been interesting to measure with an accelerometer the level of physical activity implied in the two parts of the main phase of training, which is to be considered for future studies. As future perspectives, we can regard including greater experimental criteria, as well as the possibility of working with school-age children in order to obtain health improvement in high-risk population. In this case, it would be necessary to readjust the duration of the sessions of the intervention program to the schedule of the classes of PE of Primary Education (60–90 minutes).

## 5. Conclusions

Considering the research question, it can be established that an intervention program based on active video games and motor games can improve different health indicators in university students such as fat mass, *V*O^2^_max_ or the quality of the diet. Nevertheless, this is not effective for the improvement of lean mass, flexibility or problematic use of video games. Therefore, we need to make certain points about the hypothesis:

- Hypothesis 1 (H1) was partially fulfilled, since lean mass was higher in men and lower in women, observing the opposite tendency for fat mass. In addition, the problematic use of video games was higher in men. On the contrary, the levels of resilience were higher in men, as well as the quality of the diet—not fulfilling what was established.
- Hypothesis 2 (H2) was partially fulfilled. The intervention program improved the percentage of fat mass and *V*O^2^_max_. Nevertheless, the percentage of lean mass and flexibility did not improve.
- Hypothesis 3 (H3) was partially fulfilled, since the intervention through active video games improved the quality of the diet but did not decrease the problematic use of video games.

This research study shows how an intervention program based on active video games and motor games can have slight–moderate positive effects in some of the parameters indicating physical and cognitive health in university students. It is revealed that twelve weeks of intervention enabled a slight improvement in the fat mass percentage and *V*O^2^_max_, considering the limited intensity in physiological internal load offered by these kind of devices. Additionally, some resilience factors were improved, representing a moderate size effect in confidence and tolerance for adversity, mainly due to the well-being and playful component of these devices. Finally, level of adherence to a MD was improved, due to the promotion of physical and healthy habits in the two phases of intervention.

As a main conclusion, it can be established that active video games are a resource of interest for the realization of physical exercise in the improvement of health in young adults, even though the intervention program had a low effect on most indicators because participants were young adults. Likewise, and despite the limitations described, the literature shows how this type of platform can have an even greater potential effect in children and adolescents, especially linked to the improvement of body composition and *V*O^2^_max_. This pilot study demonstrates some potential benefits of these devices, as well as its usefulness to apply some of the contents developed in the area of Physical Education. In this way, a line of research is opened in order to replicate this study in populations with a higher prevalence of overweight, such as children and adolescents.

## Figures and Tables

**Table 1:** Characteristics of the sample regarding gender prior to intervention.

Program Variables				Levene’s Test		T Test Sig. (Bilateral)
	Gender	M	SD	F	Sig.	
Body Weight	Man	74.87	11.21	0.910	0.344	0.001 *
	Woman	55.79	8.11			
BMI	Man	24.08	3.70	0.003	0.957	0.003 *
	Woman	21.09	3.41			
Fat Mass	Man	12.00	5.56	1.203	0.277	0.626
	Woman	12.75	5.91			
Lean Mass	Man	62.84	6.55	10.936	0.002	0.001 *
	Woman	43.03	2.78			
Flexibility	Man	28.91	7.54	2.322	0.133	0.578
	Woman	29.96	5.82			
*V*O^2^_max_	Man	51.12	12.79	0.301	0.586	0.001 *
	Woman	38.54	12.56			
Diet	Man	6.68	2.17	0.461	0.500	0.376
	Woman	6.09	2.81			
Video games	Man	35.94	11.01	13.178	0.001	0.001 *
	Woman	24.52	5.43			

* Statistically significant differences at level *p* < 0.05; *V*O^2^_max_, Maximum oxygen consumption (ml/min/kg); BMI, Body Mass Index (kg/m^2^); Flexibility (cm); Lean mass (%); Fat mass (%).

**Table 2:** Characteristics of the sample—resilience—regarding gender prior to intervention.

Program Variables	Gender	M	SD	Levene’s Test		T Test Sig. (Bilateral)
				F	Sig.	
F1	Man	3.45	0.39	0.574	0.452	0.200
	Woman	3.32	0.36			
F2	Man	3.05	0.25	5.538	0.022	0.231
	Woman	2.93	0.41			
F3	Man	3.45	0.41	0.387	0.536	0.175
	Woman	3.30	0.41			
F4	Man	3.27	0.43	0.623	0.433	0.906
	Woman	3.28	0.54			
F5	Man	2.63	0.66	0.966	0.330	0.394
	Woman	2.78	0.61			

F1, Personal ability and tenacity; F2, Confidence and tolerance for adversity; F3, Positive acceptance of change; F4, Control; F5, Spiritual influence.

**Table 3:** Effect of the intervention program on the variables studied.

Program Variables		M	SD	T	Sig.	d	I.C. 95%
Fat mass	(Pre-test)	12.30	5.67	2.509	0.015 *	−0.11	(−0.45–0.28)
	(Post-test)	11.83	5.28				
Lean Mass	(Pre-test)	54.85	11.15	−0.821	0.415	0.03	(−0.33–0.40)
	(Post-test)	55.22	11.09				
Flexibility	(Pre-test)	29.33	6.87	1.233	0.223	−0.11	(−0.48–0.25)
	(Post-test)	28.51	7.30				
*V*O^2^_max_	(Pre-test)	45.91	15.03	−2.767	0.008 *	0.13	(−0.28–0.46)
	(Post-test)	47.19	13.53				
RF1	(Pre-test)	3.40	0.38	−0.712	0.479	0.08	(−0.29–0.44)
	(Post-test)	3.43	0.41				
RF2	(Pre-test)	3.00	0.33	−3.360	0.001 *	0.42	(−0.02–0.76)
	(Post-test)	3.14	0.38				
RF3	(Pre-test)	3.49	0.45	−1.761	0.084	0.04	(−0.32–0.41)
	(Post-test)	3.51	0.46				
RF4	(Pre-test)	3.28	0.47	−1.373	0.175	0.14	(−0.23–0.51)
	(Post-test)	3.35	0.51				
RF5	(Pre-test)	2.69	0.64	1.427	0.159	−0.17	(−0.53–0.20)
	(Post-test)	2.58	0.68				
Video games	(Pre-test)	31.33	10.71	0.204	0.839	−0.02	(−0.38–0.35)
	(Post-test)	31.14	11.70				
Diet	(Pre-test)	6.44	2.44	−2.315	0.024 *	0.32	(−0.08–0.66)
	(Post-test)	7.11	2.20				

Note 1: F1, Personal ability and tenacity; F2, Confidence and tolerance towards adversity; F3, Positive acceptance of change; F4, Control; F5, Spiritual influence; *V*O^2^_max_, Maximum oxygen consumption (ml/min/kg); Flexibility (cm); Lean mass (%); Fat mass (%). Note 2: * Statistically significant differences at level *p* < 0.05.

**Table 4:** Bivariate correlations between variables studied after the intervention program.

Program Variables	Lean Mass	Flexibility	*V*O^2^_max_	Resilience	Video Games	Diet
Fat Mass	0.236	−0.296 *	−0.476 **	−0.023	−0.122	−0.262 *
Lean Mass		−0.258	0.323 *	0.314 *	0.424 **	0.086
Flexibility			0.142	−0.144	−0.127	0.018
*V*O^2^_max_				0.153	0.201	0.291 *
Resilience					0.083	−0.016
Video games						−0.076

* Statistically significant differences at level *p* < 0.05; ** Statistically significant differences at level *p* < 0.01.
